# Recanalization of bilateral axillaris/brachialis artery occlusion in a patient with Takayashu arteritis: First case report on using a novel drug‐coated nitinol “chocolate” balloon catheter

**DOI:** 10.1002/ccr3.1884

**Published:** 2018-11-05

**Authors:** Mariatu Binta Leigh, Samad Kor, Petar Czantrak, Mesud Sacirovic, Nikolaos Pagonas, Philipp Hillmeister, Gabriele Zeidler, Peter Bramlage, Ivo Buschmann

**Affiliations:** ^1^ Department of Obstetrics and Gynecology Vivantes Hospital Neukoelln Berlin Germany; ^2^ European Foundation for Vascular and Preventive Medicine (EFVM) Berlin Germany; ^3^ Department for Angiology, Center for Internal Medicine I Brandenburg Medical School Theodor Fontane Brandenburg Germany; ^4^ Johanniter‐Krankenhaus Treuenbrietzen Germany; ^5^ Institute for Pharmacology and Preventive Medicine Cloppenburg Germany; ^6^ DAZB (Deutsches Angiologie Zentrum Brandenburg‐ Berlin) German Center for Vascular Medicine Brandenburg‐ Berlin Germany; ^7^ Charité, Universitätsmedizin Berlin Berlin Germany

**Keywords:** drug‐coated chocolate balloon, percutaneous transluminal angioplasty, Takayasu arteritis

## Abstract

When fibrosis develops in Takayasu arteritis (TA), endovascular treatment may become necessary. A 63‐year‐old woman with TA underwent PTA with a nitinol‐structured (chocolate‐like) drug‐coated balloon (C‐DEB PTA). She remained in remission for >1 year. The case may foster research into the use of C‐DEB PTA in TA.

## 1 BACKGROUND

Takayasu arteritis (TA) is a rare chronic inflammatory disease of unknown etiology mainly affecting the aorta and its major branches such as the brachiocephalic, carotid, subclavian, vertebral, and renal arteries, as well as coronary and pulmonary arteries.^1^, ^2^ The inflammation process commences with the adventitia and progresses to the intima, and it leads to segmental stenosis, occlusion, dilatation, and/or aneurysm formation. The Chapel Hill Consensus Conference on the Nomenclature of Systemic Vasculitis classifies TA as a large‐vessel vasculitis and defines it as “granulomatous inflammation of the aorta and its major branches”.^3^ TA can lead to significant disability; more than two‐thirds of patients have trouble with their routine daily activities and up to half may be unable to work.

In the inflammatory phase of the disease, the use of prednisone‐equivalents is generally recommended.^4^ Second‐line options are cyclophosphamide, methotrexate, or cyclosporin A which are also used in case of relapse.^4^ As soon as inflammation progresses into fibrosis, endovascular (or surgical) treatment may become necessary. This may be accomplished using normal percutaneous transluminal angiography (PTA) which tends to disrupt endothelium and intima and can be associated with high rates of restenosis.^5^ For this reason, the use of paclitaxel‐eluting balloons (drug‐eluting balloon, DEB) has been suggested which resulted in less restenosis compared to simple balloon inflation.^5^, ^6^ An alternative strategy suggested the use of Scoring or Cutting Balloons resulting in controlled endothelial disruption using nitinol wires, but no drug elution.^7^

A combination of either treatment strategy has been recently become available which is a paclitaxel‐covered balloon and constrained by a nitinol structure that creates pillows and grooves in the balloon. It also provides fast deflation and uniform rewrap (chocolate drug‐eluting PTA balloon catheter (C‐DEB PTA).^8^, ^9^, ^10^, ^11^ In this case report, we describe—to the best of our knowledge—the first successful percutaneous transluminal angioplasty with a nitinol‐structured (chocolate‐like) drug‐coated (paclitaxel) balloon technology (C‐DEB PTA) in a patient with TA.

## 2 CASE PRESENTATION

We report on a 63‐year‐old female patient who presented in January 2016 with the following symptoms: complaint of cold sensation affecting the upper arms on both sides (left > right), weakness and arm claudication, with an increasing inability to perform work on the computer, and symptoms of a fatigue syndrome. The patient was unable to perform her employment as a hospital secretary. Clinically, pulselessness of the brachial and radial artery on both sides was noted. Bruits were discovered in the left and right subclavian artery. A blood test revealed increased levels of the inflammatory markers C‐reactive protein (CRP 47.9 mg/dL) and an increased Erythrocyte Sedimentation Rate (ESR 74 mm/h). The patient had a history of hypertension, hyperlipoproteinemia, and osteoporosis; she was a former smoker and reported moderate daily alcohol consumption.

A neurological examination, an MRI of the cervical spine, and myocardial scintigraphy were without pathological findings. Duplex ultrasound of the carotid artery and upper extremities revealed noticeable intima‐media thickening. For suspected large‐vessel vasculitis, a diagnostic angiography was carried out, which revealed bilateral stenosis of the axillary artery at the transition to the brachial artery and, on the left side only, an additional stenosis in the proximal segment of the brachial artery. TA was diagnosed based on the American College of Rheumatology criteria,^1^ with four out of six criteria met.

The patient was admitted to a rheumatological specialist clinic in March 2016. Treatment with prednisolone 20 mg daily was started, but there was no improvement of the arm claudication. Therefore, the patient received cyclophosphamide 15 mg/kg body weight × 0.75 (=600 mg) plus prednisolone (75 mg/d initially, reduced in stages to 30 mg/d at the time of hospital discharge). Six cycles of cyclophosphamide at 3‐week interval were planned, at the end of which treatment with methotrexate 15 mg subcutaneously once a week accompanied by a prednisolone maintenance dose of 10 mg/d was planned. Most of her symptoms regressed after initiation of the immunosuppressive therapy, but the arm claudication and weakness in the arms remained.

**Figure 1:**
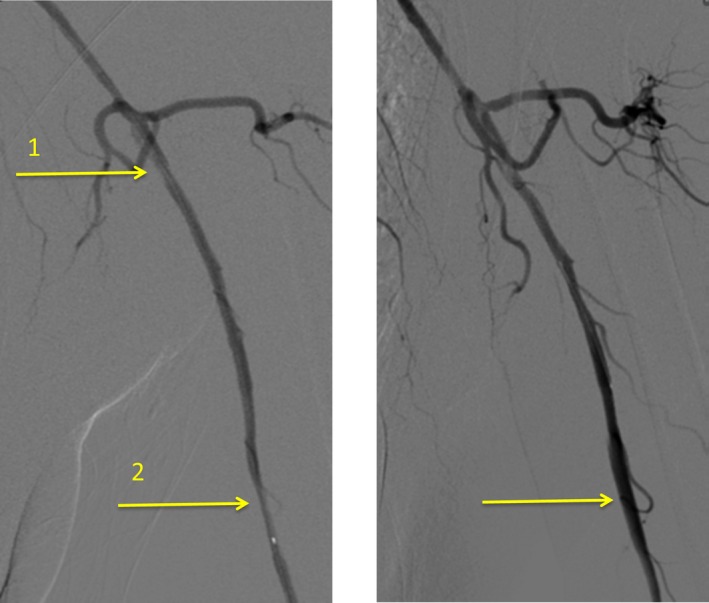
Left brachial artery before (left) and after (right) the first PTA. Left: Brachial artery with subtotal occlusion at two places: (1) moderate proximal stenosis (left axillary artery at the transition to brachial artery), (2) higher grade long‐segment stenosis (in proximal segment of left brachial artery); right: After dilatation using a 4 × 60 mm drug‐eluting Chocolate balloon (mild oversizing) of the higher grade distal stenosis

Therefore, in April 2016, she presented at our hospital for endovascular treatment. The first procedure was performed on the distal stenosis on the left side. PTA of the left proximal segment of the brachial artery was performed using a 4 × 60 mm drug‐coated Chocolate balloon (mild oversizing). The patient received a loading dose of clopidogrel 300 mg followed by a postinterventional dose of 75 mg/d orally for two months. Figure 1 shows the brachial artery with subtotal occlusion at two anatomical locations, and the artery after PTA dilatation of the distal, higher grade, stenosis. postinterventional, the patient was free of symptom on the left side. Being discharged from hospital, the patient developed mild symptoms on the left side again, although they were significantly milder than before the intervention. Moreover, the patient received a cumulative cyclophosphamide dose of 3.6 g (4 of 6 cycles).

**Figure 2:**
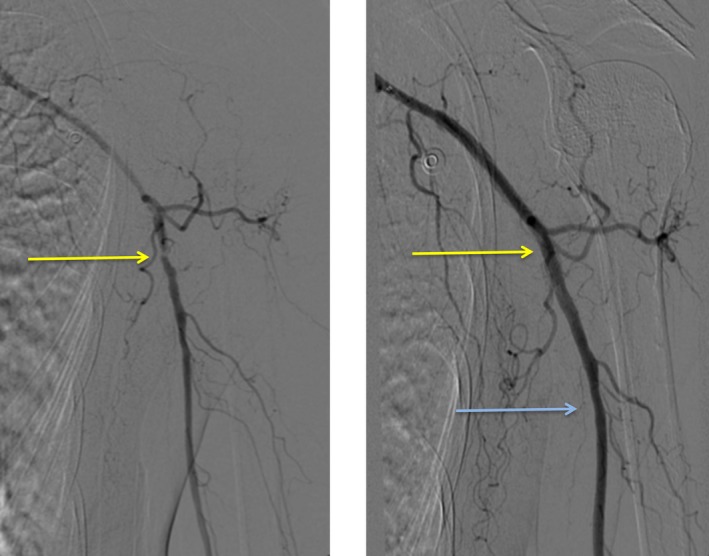
Left axillary and brachial artery before (left) and after (right) the second PTA. Left: Axillary artery at the transition to brachial artery now presenting as a high‐grade stenosis; right: After dilatation with a 4 × 60 mm drug‐eluting Chocolate balloon. The blue arrow shows the very good result of the first PTA

In June 2016, a repetitive C‐DEB PTA of the proximal stenosis on the left side was performed. This arterial segment had not been treated in the first intervention. However, due to the excellent distal result, C‐DEB PTA was performed with a 4 × 60 mm drug‐eluting Chocolate balloon on the remaining stenosis of the left axillary artery at the transition to the brachial artery, which had previously been moderate but was now presenting as higher grade (Figure 2). Treatment with clopidogrel 75 mg/d was continued. The procedure resulted in complete disappearance of arm claudication on the left side; the patient reported that she was able to use her left arm without restriction or complaints.

**Figure 3:**
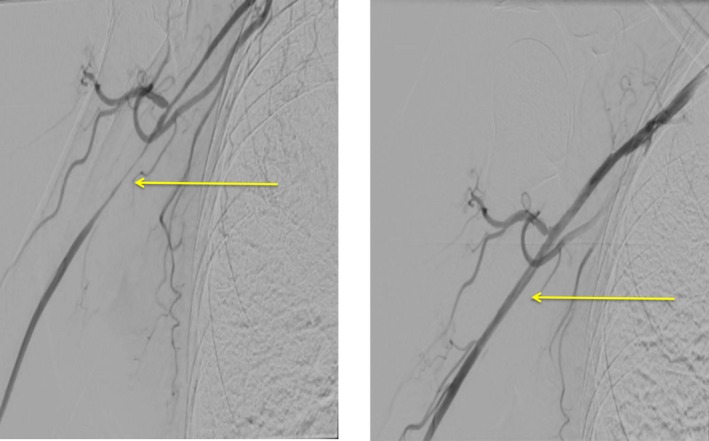
Right axillary artery before (left) and after (right) the PTA. Left: Right axillary artery presents a subtotal long‐segment stenosis up to the transition to the brachial artery; right*:* After dilatation with a 5 × 200 mm drug‐eluting Chocolate balloon

**Figure 4:**
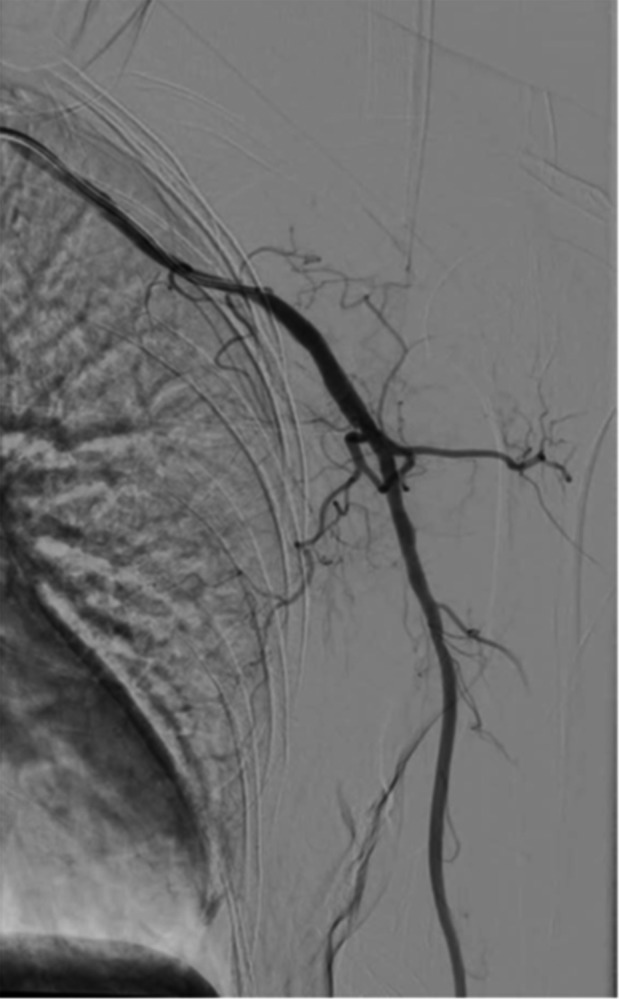
Results of the two PTAs on the left side at the time of the third intervention. The results of the two PTAs on the left side were confirmed by angiography during the intervention on the right arm

Consequently, it was decided to also perform the endovascular intervention on the right‐sided stenosis. By August 2016, the patient had completed all cyclophosphamide cycles (cumulative total dose of 5.6 g), and the medication plan consisted of methotrexate 15 mg subcutaneously once a week plus prednisolone 10 mg/d and acetylsalicylic acid 100 mg/d orally. PTA with a 5 × 200 mm drug‐eluting balloon was performed on the stenosis of the right axillary artery at the transition to the brachial artery, which had previously been moderate but now presented as higher grade (Figure 3). The successful effect of the previous two interventions on the left side was also confirmed at this time (Figure 4). Medical treatment with clopidogrel 75 mg/d was continued. After this intervention, the patient was free of claudication symptoms and was able to perform her daily work. The treatment with methotrexate, prednisolone, and acetylsalicylic acid was continued.

The patient remained in remission until the last follow‐up visit, in October 2017. A color‐coded duplex sonographic flow profile was normal, with no evidence of pathology. As a result of the intervention, the patient felt very comfortable with the clinical result of her treatment. As a cobenefit, she could escape early retirement.

## 3 DISCUSSION

Repeated PTA with a drug‐eluting Chocolate balloon catheter was successfully performed in this case of a woman suffering from a Takayasu arteritis. The case highlights several points of interest.

The latest, 2010 guideline recommendations of the *German Society of Vascular Surgery* on the treatment of outgoing/branch stenosis and occlusions of the aortic arch branches advises against endovascular revascularization in the context of occlusive processes due to inflammatory disease.^4^ The recommendations argue that: (a) Even after drug pretreatment, high rates of reocclusion and other complications are reported if (conventional balloon) PTA is performed during inflammatory remission or control. (b) Available data on endovascular treatments are based on case reports or case series with low patients numbers. The underlying evidence has changed since and with the nitinol‐structured (chocolate‐like) drug‐coated (paclitaxel) balloon technology (C‐DEB PTA), new technology has become available.^8^, ^9^ A literature search on PubMed performed on September 23rd 2018 revealed only eight references based on the keywords “chocolate” and “balloon.” In combination with “Takayasu,” no reference is left. As such, the current results need to be discussed based in a wider context.

So far, there has been no evaluation of the data relating to the results of drug‐eluting balloon therapy. However, case reports with good results have been published.^6^, ^12^ Spacek et al^6^ describe the case of a 58‐year‐old woman with TA whose carotid stenosis was initially treated with bare metal stents. The patient suffered from recurring localized stenosis. Therefore, she was treated with a coronary drug‐eluting stent and drug‐eluting balloon, resulting in a favorable long‐term outcome. Kazibudzki et al^12^ performed successful treatment of a highly symptomatic patient with multivessel TA using drug‐eluting stents and balloons. The 33‐year‐old women showed good immediate and mid‐term results. The outcomes of these cases are consistent with the case presented here, in which PTA with a drug‐coated balloon provided good results in a patient with stenoses of the axillary and brachial arteries.

The appropriate technique for performing balloon inflation during angioplasty is very important for the result.On the one hand, underinflation could potentially lead to elastic recoil, on the other hand, overinflation could lead to neointimal hyperplasia. Both could result in restenosis.^13^ To achieve the best possible treatment results with angioplasty, it is important to minimize the strain on the vessel wall. If a standard plain angioplasty balloon unfolds during inflation, an unequal application of force is applied to the stenotic lesion. Uncontrolled expansion has the potential to generate increased torsional, longitudinal, and radial stresses to the vessel wall, which could lead to dissection, elastic recoil, and abrupt vessel closure. In contrast, controlled dilatation can help mitigate these stresses. To this end, the Chocolate DEB‐ PTA balloon catheter was developed.^13^ It has a mounted nitinol constraining structure which helps to protect the vessel from pathological shear forces caused by balloon inflation. This retaining structure results in balloon pillows and grooves; the pillows enable vessel dilatation without cutting or scoring, and the grooves provide stress relief and plaque modification.

In the Chocolate Balloon Angioplasty Registry (BAR) (NCT01589042),^10^ procedural success was reported for 85.1% of cases, and freedom from stenting occurred in 93.1%. Bailout stenting by independent adjudication occurred in 1.6% of cases, and there were no flow‐limiting dissections. The dissection rate as well as the rate of bailout stenting was much than reported for standard PTA balloons. Another trial conducted with a Chocolate balloon catheter is the completed, but not yet fully reported ENDURE trial (NCT02129127: Drug‐Coated PTA Balloon in Patients with Peripheral Arterial Disease). This first‐in‐man study evaluated the drug‐coated Chocolate balloon for percutaneous arterial angioplasty in patients with symptomatic peripheral arterial disease. The study focuses on acute device performance and peri‐procedural safety and also seeks to further characterize other aspects of the performance of the device. Initial results were presented at the Leipzig Interventional Course in January 2017.^14^ The drug‐coated Chocolate Touch achieved a low residual diameter stenosis (>50% diameter stenosis 1.4%) and no flow‐limiting dissections, resulting in an extremely low rate of per‐protocol bailout stenting (1.4%). Promising evidence of the drug effect was shown by way of 12‐month patency and low late lumen loss at 6 months.

Clinical Implications: This case raises several general points about the management of patients with TA. (a) To date, no long‐term data comparing the Chocolate drug‐eluting balloon with other drug‐eluting balloons are available. (b) Prospective registries are necessary to document time course of disease, conservative, and/or endovascular treatment as well as their respective outcomes. (c) This might provide evidence for a guideline adjustment in the near future.

## 4 CONCLUSION

In a 63‐year‐old female patient with TA and bilateral axillaris/brachialis, artery occlusion repeated C‐DEB PTA with a drug‐eluting Chocolate balloon catheter provided a successful clinical outcome. Further evaluation of this interventional treatment modality in larger patient populations is warranted to help optimize treatment for patients with TA.

## CONSENT

Informed consent was obtained from the patient for publication of this case report and accompanying images. A copy of this is available from the corresponding author.

## CONFLICT OF INTEREST

The authors declare that they have no competing interests.

## AUTHOR CONTRIBUTIONS

IB together with MBL: had the conceptual idea. SK, PC, MS, NP, PH and GZ: were involved in the clinical treatment of the patient. PB and MBL: wrote the initial draft of the manuscript which was revised by all authors for important intellectual content. All authors approved the final content and take over responsibility for the content of the manuscript.
